# Association of platelet count with all-cause mortality and risk of cardiovascular and respiratory morbidity in stable COPD

**DOI:** 10.1186/s12931-019-1059-1

**Published:** 2019-05-08

**Authors:** Ashraf Fawzy, Julie A. Anderson, Nicholas J. Cowans, Courtney Crim, Robert Wise, Julie C. Yates, Nadia N. Hansel

**Affiliations:** 1 Division of Pulmonary and Critical Care Medicine, Johns Hopkins University, 1830 E. Monument St. 5th Floor, Baltimore, MD USA; 2 Research & Development, GlaxoSmithKline plc, Stockley Park, Middlesex, UK; 3 Statistics and Programming, Veramed Ltd, Twickenham, UK; 4 Research & Development, GlaxoSmithKline plc, Research Triangle Park, NC USA

**Keywords:** Platelet count, Chronic obstructive pulmonary disease, Mortality, Cardiovascular disease, Exacerbations

## Abstract

**Background:**

Platelet count is a prognostic indicator in the general population and elderly. Thrombocytosis during acute exacerbation of COPD (AECOPD) has been associated with mortality; however, the relationship between platelet count and mortality in stable COPD is unknown.

**Methods:**

We performed post hoc secondary analysis on a subsample of 1797 patients in the Study to Understand Mortality and Morbidity in COPD (SUMMIT) who had blood samples drawn at baseline. Participants were current or former smokers, 40–80 years old with moderate COPD and history or increased risk of cardiovascular (CV) disease. The primary outcome was on and post-treatment all-cause mortality. Secondary outcomes included first-on-treatment moderate/severe AECOPD and on-treatment CV composite event (CV death, myocardial infarction, stroke, unstable angina and transient ischemic attack). Multivariable Cox proportional hazards models were used to investigate study endpoint associations with platelet count quintile grouping, continuous platelet count utilizing two-term fractional polynomials, and categories of low, normal and high platelet count (< 150, ≥150 to < 300, ≥300 × 10^9^/L).

**Results:**

Patients were followed for 2.3 ± 0.9 years for vital status and 1.6 ± 1.1 years for morbidity endpoints during which 105 (5.8%) died, 651 (36.2%) experienced AECOPD (159 with severe AECOPD) and 86 (4.8%) experienced a CV event. A U-shaped association between platelet count and all-cause mortality was observed. Compared to the third quintile group (Q3) of platelet count, risk of death was increased in the lowest quintile group (Q1; hazard ratio [HR]: 1.73; 95% confidence interval [CI]: 0.93–3.23) and highest quintile group (Q5; HR: 1.66; 95%CI: 0.89–3.10), though point estimates were imprecise. Using clinical cutoffs, compared with normal platelet counts (≥150 to < 300 × 10^9^/L), risk of all-cause mortality was nominally increased among patients with thrombocytopenia (HR: 1.46; 95%CI: 0.81–2.64) and high platelet count (HR: 1.66; 95%CI: 0.96–2.86). Compared with Q3, CV events were nominally increased for Q5 (HR: 1.71; 95%CI: 0.83–3.49) and Q1 (HR: 1.41; 95%CI: 0.70, 2.85). There was no association between platelet count and AECOPD.

**Conclusions:**

In stable COPD platelet count demonstrated a U-shaped association with increased risk of 3-year all-cause mortality, though a platelet count level above or below which risk of mortality was increased could not be definitively identified.

**Trial registration:**

ClinicalTrials.gov NCT01313676.

## Background

Chronic obstructive pulmonary disease (COPD) is projected to become the third leading cause of death by 2030, representing 8.6% of global deaths [1]. Chronic systemic inflammation in COPD has been associated with poor clinical outcomes [2, 3]. Biomarkers have potential value as prognostic indicators in COPD, though their utility has been limited by disease heterogeneity and the influence of comorbidities [4]. Platelet count, a component of a routinely measured clinical assay, remains within a relatively narrow range among healthy individuals but can be significantly altered in the setting of both acute and chronic disease [5]. Notably, platelet count is correlated with levels of fibrinogen [6], an inflammatory biomarker identified as a means of selecting research participants with COPD at high risk for morbidity and mortality [7, 8]. A U-shaped association between platelet count and increased mortality has been recognized in the general population and the elderly [9–14], though its role in COPD remains unclear. In addition to the well documented role of platelets in cardiovascular (CV) disease, platelets are now recognized as playing a role in many pathophysiologic processes such as inflammation, host defense, and tumor biology [15].

Overall, platelet count in COPD has been shown to be elevated compared with the general population [16, 17]. Previous studies have demonstrated an increased risk of in-hospital and 1-year mortality associated with thrombocytosis (defined as platelet count > 400 × 10^9^/L) measured at the time of acute exacerbation of COPD (AECOPD) [18]. In univariate analysis, thrombocytopenia measured on admission for AECOPD was also associated with in-hospital mortality, need for mechanical ventilation, intensive care unit admission, and length of hospitalization [19]. Our group has recently demonstrated that elevated platelet count (> 350 × 10^9^/L) measured in stable COPD is associated with higher likelihood of prior exacerbation and worse respiratory symptoms [20]. However, the relationship between platelet count measured in the stable phase of COPD and all-cause mortality has not been previously explored. Furthermore, comorbid COPD and CV disease has been associated with higher rates of morbidity than presence of either disease alone making this a particularly vulnerable population [21]. Using data from the Study to Understand Mortality and Morbidity in COPD (SUMMIT), a prospective randomized controlled trial enriched for presence of CV comorbidity and containing adjudicated events, we investigated post hoc the association of platelet count measured in the stable phase of COPD with all-cause mortality, CV and respiratory outcomes.

## Methods

### Study population

SUMMIT was a prospective, multi-center, international randomized controlled trial to determine whether treatment with an inhaled long-acting beta-agonist (LABA) in combination with an inhaled corticosteroid (ICS), versus either component, could improve clinical outcomes in patients with moderate COPD and increased CV risk compared with placebo. Details regarding study design have been previously published [22, 23]. In brief, participants were required to be current or former smokers (≥10 pack-years) between the ages of 40 and 80 years, with a history of moderate COPD defined as post-bronchodilator forced expiratory volume in 1 s (FEV_1_) between 50 and 70% of the predicted value, a ratio of post-bronchodilator FEV_1_ to forced vital capacity ≤0.70, and a score ≥ 2 on the modified Medical Research Council (mMRC) dyspnea scale. Patients were additionally required to have a history, or be at increased risk, of CV disease. CV disease was defined as coronary artery disease, peripheral arterial disease, prior stroke or myocardial infarction, or diabetes mellitus with target organ disease. Increased CV risk was defined as being ≥60 years and receiving medications for two or more of the following: hypercholesterolemia, hypertension, diabetes mellitus or peripheral vascular disease.

While prior ICS and LABA treatments were discontinued before study entry, other COPD medications were permitted during the trial. Participants were then allocated equally to one of four randomized treatments: placebo, fluticasone furoate (100 μg), vilanterol (25 μg) or their combination (fluticasone furoate/vilanterol, 100/25 μg) inhaled once daily as a dry powder. A total of 16,485 patients were enrolled and included in the final intention-to-treat efficacy population. This secondary analysis included a subsample of 1797 patients who had blood samples drawn, all of whom were recruited in the United States.

### Platelet count

Venous blood samples were obtained before randomization and at 3 months. Blood was processed, and plasma stored at − 80 °C until analyzed. Platelet counts were performed by Q^2^ Solutions (Valencia, CA). Only baseline platelet count was used in this analysis to avoid survivorship bias and ensure that platelet count is not affected by competing outcomes.

### Outcomes

The primary outcome of interest is on- and post-treatment all-cause mortality, with secondary outcomes including first-on-treatment AECOPD or on-treatment CV event defined as a composite of CV death, myocardial infarction, stroke, unstable angina and transient ischemic attack [24]. Moderate AECOPD was defined as a symptomatic deterioration requiring treatment with antibiotic drugs or systemic corticosteroids, whereas severe AECOPD was defined as an event leading to hospital admission. Categorization of the cause of each death was adjudicated by a clinical endpoint committee blinded to the treatment allocation who also determined whether any reported CV event met the definition of the composite endpoint [22]. Follow-up visits were not performed after a patient discontinued study treatments therefore the only complete data for those patients was regarding mortality. Data on CV events and COPD exacerbations were no longer collected.

### Statistical analysis

Patients were split into quintile groups based on their baseline platelet count and patient characteristics were summarized.

To explore the effect of baseline platelet count quintile on each of the study endpoints (all-cause mortality, CV composite, moderate/severe AECOPD and severe AECOPD), analysis of time-to-first event was performed using Cox proportional hazards regression modelling, using the middle quintile group as the reference. In the mortality analysis participants who did not die were censored at the end of the study, or when they were lost to follow-up if this occurred first. For all other analyses patients were censored if they discontinued use of study treatments or were lost to follow-up. Models were adjusted for age, sex, race, ethnicity, BMI category, smoking status, percent predicted FEV_1_, previous history of AECOPD, anemia (hematocrit < 36% for women and < 39% for men), SUMMIT CV entry criteria (aged < 60 with CV disease, aged ≥60 with CV disease, aged ≥60 with CV risk), ischemic and vascular disease indicators (e.g., previous treatment of coronary or vascular disease), mMRC score, and study treatment. Sensitivity analysis including use of antiplatelet therapy as a covariate was performed for all outcomes.

Baseline platelet count was also examined as a continuous variable where the best fitting model was selected from a variety of polynomial or logarithmic models using two-term fractional polynomials [25]. As a secondary analysis, patients were regrouped into three categories of low, normal and high platelet count (< 150, ≥150 to < 300, and ≥ 300 × 10^9^/L) based on a standard clinical definition of thrombocytopenia and a platelet count threshold above which previous reports in the general population have identified an increased mortality risk [9, 11–13].

Statistical analyses were performed using SAS 9.4 (Carey, NC). All patients provided written informed consent. The study was approved by local ethics committees and was conducted in accordance with the Declaration of Helsinki and Good Clinical Practice guidelines. Scientific oversight of the trial was provided by a steering committee composed of academic experts and employees from GlaxoSmithKline plc, who were collectively responsible for the study design and analysis, and for the review and interpretation of the data. This study is registered with ClinicalTrials.gov, number NCT01313676.

## Results

Among the subgroup of 1797 participants with platelet count measurement median platelet count was 219 × 10^9^/L (interquartile range [IQR] 181–260 × 10^9^/L). One participant with platelet count 1415 × 10^9^/L was excluded from analysis. Baseline characteristics are presented in Table 1 for the entire cohort and by platelet count quintile group (Q1: < 173 × 10^9^; Q2: ≥173 to < 205 × 10^9^; Q3: ≥205 to < 234 × 10^9^; Q4: ≥234 to < 272 × 10^9^; Q5: ≥272 × 10^9^). The higher platelet count groups included more females of younger average age with lower prevalence of obesity and cardiac comorbidities but higher prevalence of anemia and current smoking.

**Table 1:** Patient characteristics in the platelet population and split by baseline platelet count quintile. Mean ± standard deviation or count (%) shown

Characteristics	Whole Sample (*N* = 1796)	Platelet Count Quintile Group 1: < 173 (*N* = 363)	Platelet Count Quintile Group 2: > = 173 to < 205 (*N* = 356)	Platelet Count Quintile Group 3: > = 205 to < 234 (*N* = 354)	Platelet Count Quintile Group 4: > = 234 to < 272 (*N* = 364)	Platelet Count Quintile Group 5: > = 272 (*N* = 359)
Age, years	66 ± 8	67 ± 7	66 ± 8	66 ± 8	65 ± 8	65 ± 8
Female	683 (38%)	65 (18%)	108 (30%)	148 (42%)	170 (47%)	192 (53%)
Race						
White	1608 (90%)	334 (92%)	316 (89%)	319 (90%)	328 (90%)	311 (87%)
Asian	22 (1%)	5 (1%)	5 (1%)	3 (< 1%)	1 (< 1%)	8 (2%)
Other	166 (9%)	24 (7%)	35 (10%)	32 (9%)	35 (10%)	40 (10%)
Body Mass Index (BMI), kg/m^**2**^	31 ± 7	31 ± 6	31 ± 7	30 ± 7	31 ± 7	29 ± 7
Underweight (BMI < 18.5 kg/m^**2**^)	30 (2%)	5 (1%)	5 (1%)	7 (2%)	4 (1%)	9 (3%)
Normal/Overweight (BMI 18.5–30 kg/m^**2**^)	878 (49%)	157 (43%)	159 (45)	178 (50)	188 (52%)	196 (54%)
Obese (BMI ≥ 30 kg/m2)	888 (49%)	201 (55%)	192 (54%)	169 (48%)	172 (47%)	154 (43%)
FEV_1_, % predicted	59 ± 7	59 ± 7	60 ± 7	59 ± 7	60 ± 7	59 ± 7
Post-bronchodilator FEV_1_, L	1.7 ± 0.4	1.8 ± 0.4	1.8 ± 0.4	1.7 ± 0.4	1.7 ± 0.4	1.6 ± 0.4
Current Smokers	891 (50%)	170 (47%)	176 (49%)	175 (49%)	184 (51%)	186 (52%)
Pack years smoked	52 ± 29	55 ± 31	52 ± 28	51 ± 30	53 ± 29	50 ± 27
Oxygen Therapy	78 (4%)	18 (5%)	10 (3%)	14 (4%)	21 (6%)	15 (4%)
Hematocrit, %	43 ± 5	44 ± 5	44 ± 4	43 ± 4	42 ± 4	41 ± 5
Anemia: Hematocrit < 39% (men) or < 36% (women)	212 (12%)	38 (10%)	34 (10%)	34 (10%)	45 (12%)	61 (17%)
Previous medical history						
Hypertension	1630 (91%)	342 (94%)	326 (92%)	312 (88%)	320 (88%)	330 (92%)
Coronary arterial disease	877 (49%)	211 (58%)	195 (55%)	162 (46%)	161 (44%)	148 (41%)
Congestive heart failure	159 (9%)	38 (10%)	36 (10%)	29 (8%)	33 (9%)	23 (6%)
Stroke	138 (8%)	20 (6%)	27 (8%)	27 (8%)	32 (9%)	32 (9%)
Diabetes	697 (39%)	158 (44%)	138 (39%)	129 (36%)	141 (39%)	131 (36%)
Pre-study COPD therapy						
Inhaled Corticosteroid	860 (48%)	172 (47%)	155 (44%)	187 (53%)	163 (45%)	183 (51%)
Long-acting anticholinergic	542 (30%)	107 (29%)	94 (26%)	110 (31%)	110 (30%)	121 (34%)
Long-acting beta-2 agonist	802 (45%)	161 (44%)	144 (40%)	170 (48%)	154 (42%)	173 (48%)
Concomitant cardiovascular therapy						
Any Medication	1755 (98%)	360 (> 99%)	351 (99%)	345 (97%)	354 (97%)	345 (96%)
Antithrombotic/coagulant	1225 (68%)	265 (73%)	264 (74%)	238 (67%)	228 (63%)	230 (64%)
Antiplatelet therapy	1156 (64%)	246 (68%)	248 (70%)	223 (63%)	220 (60%)	219 (61%)
Statins	1362 (83%)	282 (78%)	274 (77%)	271 (77%)	274 (75%)	261 (73%)
Beta-blockers	837 (47%)	197 (54%)	182 (51%)	153 (43%)	162 (45%)	143 (40%)
Randomization Group						
Placebo	469 (26%)	79 (22%)	88 (25%)	115 (32%)	96 (26%)	91 (25%)
Fluticasone Furoate	449 (25%)	94 (26%)	87 (24%)	87 (25%)	93 (26%)	88 (25%)
Vilanterol	436 (24%)	101 (28%)	88 (25%)	65 (18%)	89 (24%)	93 (26%)
Fluticasone + Vilanterol	442 (25%)	89 (25%)	93 (26%)	87 (25%)	86 (24%)	87 (24%)

Patients were on study treatment for an average of 1.6 (± 1.1 SD) years for a total of 3848 person-years, during which time 86 (4.8%) experienced a CV event and 651 (36.2%) experienced an AECOPD, of which 159 (24.4%) subjects experienced a severe AECOPD. Patients were followed-up on and post treatment for an average of 2.3 ± 0.9 years for vital status during which time 105 (5.8%) participants died (Table 2). Four participants were lost to follow-up and did not have vital status recorded at the end of the study.

**Table 2:** (A) Time to all-cause death (and breakdown by adjudicated cause) and time to first (B) moderate/severe COPD exacerbation, (C) severe COPD exacerbation and (D) cardiovascular (CV) composite event by baseline platelet count

Event	Platelet Count Quintile Group 1: < 173 (N = 363)	Platelet Count Quintile Group 2: > = 173 to < 205 (N = 356)	Platelet Count Quintile Group 3: > = 205 to < 234 (N = 354)	Platelet Count Quintile Group 4: > = 234 to < 272 (N = 364)	Platelet Count Quintile Group 5: > = 272 (N = 359)
(A)					
All-cause Mortality	30 (8.3%)	17 (4.8%)	16 (4.3%)	13 (3.7%)	30 (8%)
Hazard Ratio (95% CI), vs. Quintile group 3	1.73 (0.93, 3.23)	0.97 (0.48, 1.96)	-REF-	0.77 (0.37, 1.61)	1.66 (0.89, 3.10)
Cause Specific Death					
Respiratory	6 (20%)	2 (12%)	1 (6%)	1 (8%)	2 (7%)
Cardiovascular	6 (20%)	5 (29%)	6 (38%)	6 (46%)	9 (30%)
Cancer	11 (37%)	5 (29%)	4 (25%)	4 (31%)	7 (23%)
Other	5 (17%)	2 (12%)	1 (6%)	0 (0%)	3 (10%)
Unknown	2 (7%)	3 (18%)	4 (25%)	2 (15%)	9 (30%)
(B)					
Moderate/Severe COPD Exacerbation	129 (36%)	115 (32%)	132 (37%)	128 (35%)	147 (41%)
Hazard Ratio (95% CI), vs. Quintile group 3	0.91 (0.71, 1.17)	0.82 (0.63, 1.06)	-REF-	0.87 (0.68, 1.12)	1.00 (0.79, 1.28)
(C)					
Severe COPD Exacerbation	35 (10%)	25 (7%)	27 (8%)	33 (9%)	39 (11%)
Hazard Ratio (95% CI), vs. Quintile group 3	1.16 (0.69, 1.95)	1.00 (0.57, 1.74)	-REF-	1.20 (0.71, 2.02)	1.33 (0.80, 2.21)
(D)					
CV Composite Event	23 (6%)	15 (4%)	13 (4%)	15 (4%)	20 (6%)
Hazard Ratio (95% CI), vs. Quintile group 3	1.41 (0.70, 2.85)	1.11 (0.52, 2.37)	-REF-	1.14 (0.53, 2.45)	1.71 (0.83, 3.49)

Participants in the highest quintile group of platelet count had the highest proportion of moderate/severe AECOPD (41%) and severe AECOPD (11%). CV composite events (6%) and deaths (8%) in the highest quintile were equivalent to the lowest quintile of platelet count but higher than the middle quintiles.

### Association of platelet count with mortality

**Fig. 1:**
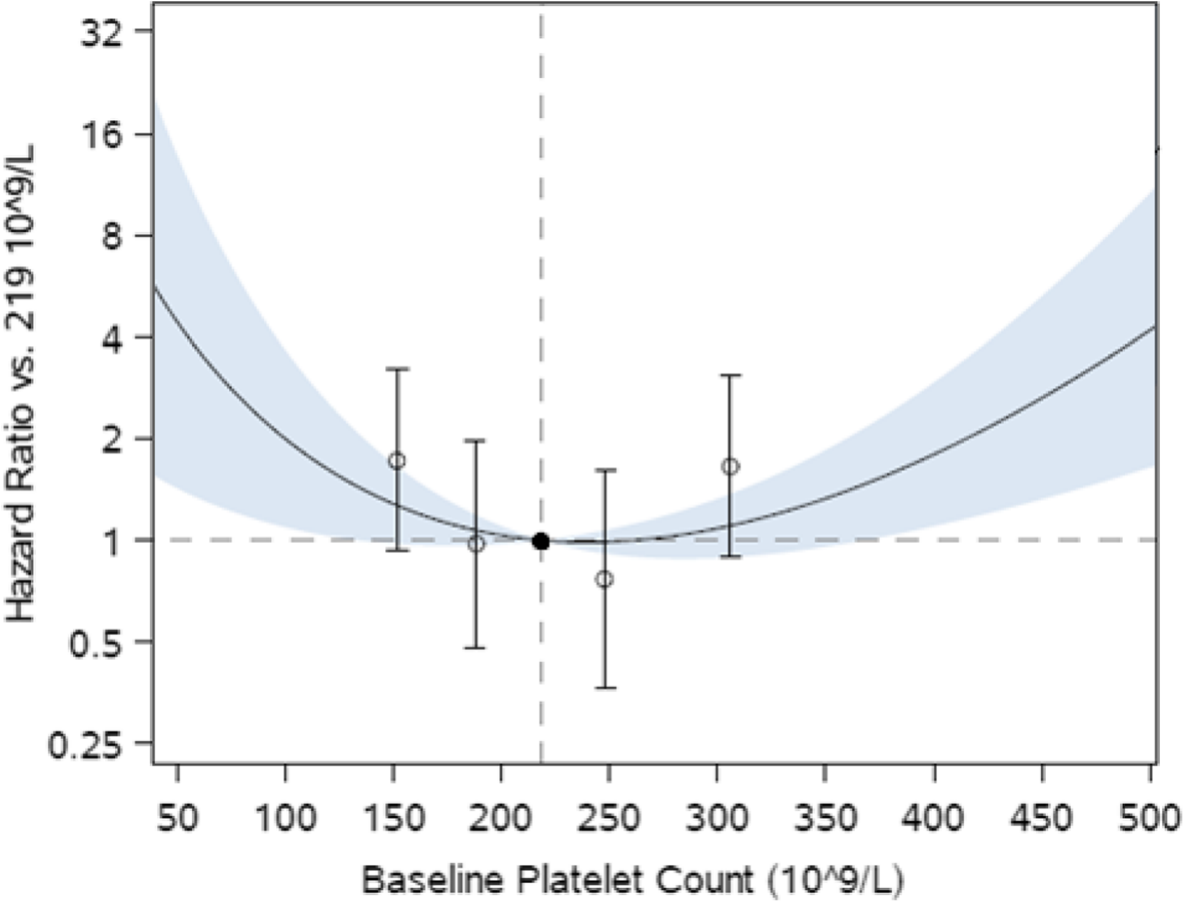
Association of platelet count with death from any cause. Open circles and 95% confidence intervals represent hazard ratio in each quintile in reference to middle quintile, shown as a filled circle. Curve and shaded region show hazard ratio and 95% confidence region of platelet count as a continuous variable in reference to the median platelet count

In multivariable analysis a U-shaped association between platelet count and all-cause mortality was observed (Fig. 1). Compared with the third quintile (Q3) of platelet count, patients in the lowest quintile (Q1) had a 73% increased risk of death (95% confidence interval [CI]: − 7 to 223%) while patients in the highest quintile (Q5) had a 66% increased risk of death (95% CI: -11 to 210%), though these estimates were imprecise. Cause-specific mortality by platelet count quintile is presented in Table 2.

When using clinical ranges, compared with patients who had platelet counts of 150-300 × 10^9^/L, those with thrombocytopenia (< 150 × 10^9^/L) had a 46% increased risk of death (95% CI: -19 to 164%) and those with high platelet count (≥300 × 10^9^/L) had a 66% increased risk (95% CI: -4 to 186%); however, similar to quintile analyses, point estimates were imprecise.

### Association of platelet count with CV and respiratory endpoints

**Fig. 2:**
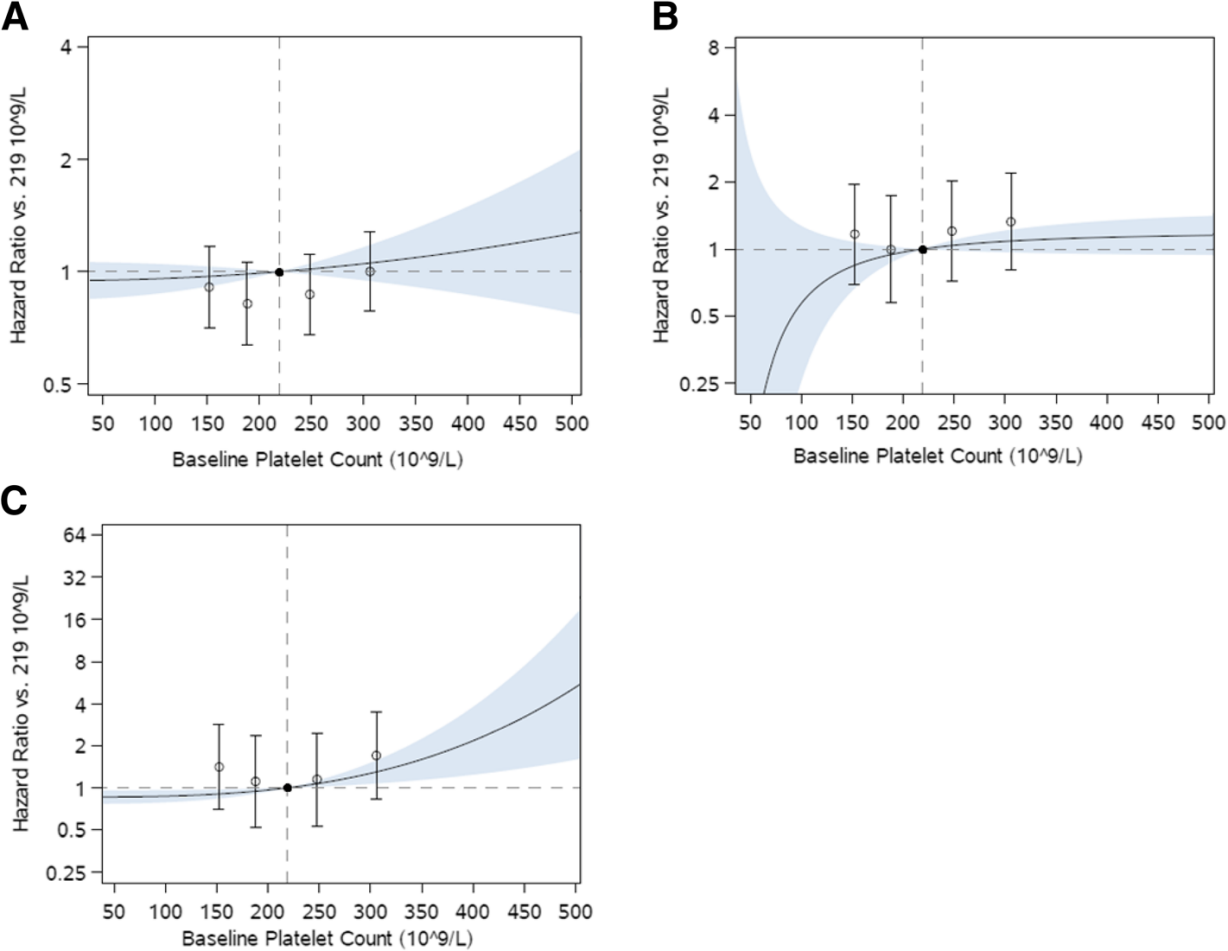
Association of platelet count with first on-treatment (**a**) moderate/severe COPD exacerbations, **b** severe COPD exacerbations, and (**c**) cardiovascular composite event (cardiovascular death, myocardial infarction, stroke, unstable angina, and transient ischemic attack) Open circles and 95% confidence intervals represent hazard ratio in each quintile in reference to middle quintile, shown as a filled circle. Curve and shaded region show hazard ratio and 95% confidence region of platelet count as a continuous variable in reference to the median platelet count

Higher platelet counts also were associated with nominally increased hazard of a CV composite event when analyzed as platelet count quintiles (Q5 vs. Q3 hazard ratio [HR]: 1.71; 95% CI: 0.83–2.49). Analyses using clinical ranges showed that, compared with normal platelet count (≥150 to < 300 × 10^9^/L), elevated platelet count (≥300 × 10^9^/L) was associated with 55% increased risk of composite CV outcome (95% CI: -18 to 194%), though this association was also not statistically significant. Compared with Q3, patients in Q1 had nominally increased composite CV events (HR: 1.41; 95% CI: 0.70, 2.85), though this was not consistent with results of secondary analyses (Fig. 2, Table 3). There was no significant association between platelet count and AECOPD, both moderate/severe and severe (Fig. 2, Tables 2 and 3). Sensitivity analyses including use of antiplatelet therapy as an additional covariate produced equivalent results for all outcomes (data not shown).

**Table 3:** Number of subjects having an event (%) and hazard ratio (95% confidence interval) of time to all-cause death, moderate/severe COPD exacerbation, severe COPD exacerbation, and cardiovascular composite event among participants with thrombocytopenia (platelet count < 150 × 10^9^ /L) and high platelet count (≥ 300 × 10^9^ /L) compared with normal platelet count (≥150 to < 300 × 10^9^ /L)

Event	Platelet count < 150 × 10^9^ /L *N* = 167	Platelet count ≥150 × 10^9^ /L to < 300 × 10^9^ /L *N* = 1424	Platelet count ≥300 × 10^9^ /L *N* = 206
All-cause death	14 (8%)	73 (5%)	19 (9%)
	1.46 (0.81, 2.64)	-REF-	1.66 (0.96, 2.86)
Cardiovascular composite	6 (4%)	68 (5%)	12 (6%)
	0.55 (0.23, 1.30)	-REF-	1.55 (0.82, 2.94)
Moderate/Severe COPD exacerbation	64 (38%)	508 (36%)	79 (38%)
	1.15 (0.88, 1.50)	-REF-	1.05 (0.82, 1.34)
Severe COPD exacerbation	15 (9%)	123 (9%)	21 (10%)
	0.95 (0.55, 1.66)	-REF-	1.21 (0.75, 1.96)

## Discussion

In this prospective study of patients with COPD and prior history or increased risk of CV disease platelet count measured in the stable state demonstrated a U-shaped association with increased risk of 3-year all-cause mortality, though a platelet count level above or below which risk of mortality was increased could not be definitively identified. Higher platelet count also showed trends towards increased risk for a composite outcome of CV morbidity and mortality though these results did not reach statistical significance. Platelet count did not appear to be associated with prospective risk for AECOPD. To our knowledge, this is the first study investigating the association between platelet count measured in the stable state of COPD and all-cause mortality. These findings expand upon prior studies reporting increased all-cause mortality among COPD patients with thrombocytosis and thrombocytopenia measured during an AECOPD [18, 19].

The U-shaped association between platelet count and mortality observed in this sample of stable COPD patients has been previously described in a general adult population [13, 14], postmenopausal women [9], and the elderly [10–12]. Increased risk of all-cause mortality was consistently noted in these prior studies for platelet count > 300 × 10^9^/L but not thrombocytopenia (< 150 × 10^9^/L) [11–13]. While higher platelet counts appeared to be associated with increased mortality in this study of COPD patients, platelet count ≥300 × 10^9^/L and thrombocytopenia trended toward increased risk of mortality, though did not reach statistical significance. This may be a consequence of a relatively small overall and group specific sample sizes compared with studies of non-COPD cohorts or risk for mortality present only at higher platelet counts, similar to those identified in prior studies of mortality following AECOPD (> 400 × 10^9^/L) and respiratory symptoms (≥350 × 10^9^/L, which we did not have adequate power to evaluate [18, 20].

Elevated platelet count has been associated with CV mortality in healthy middle-aged men but not the elderly [11, 12, 26], and with a composite CV outcome of myocardial infarction, cerebrovascular disease, or peripheral vascular disease in the general population [13]. Though we were not able to evaluate CV mortality independently due to low event rate, higher platelet count, evaluated as a continuous variable, appeared associated with a composite CV outcome in this sample of COPD patients with history or increased risk of CV disease, though higher platelet count categories did not reach statistical significance. Notably, elevated platelet count was not associated with AECOPD in this study which is in contrast to a prior study demonstrating association between platelet count ≥350 × 10^9^/L measured in the stable state and report of at least one AECOPD in the prior year [20]. Thus, it appears that platelet count may not inform risk of future AECOPD beyond prior exacerbation history. Additionally, in this higher risk sample of stable COPD patients with comorbid CV disease, platelet count appears to be a predictor of CV rather than respiratory morbidity.

Potential biological mechanisms for the association of elevated platelet count with all-cause mortality and CV morbidity in COPD include systemic inflammation, atherosclerotic plaque destabilization, and platelet activation. Previous studies investigating the prognostic value of biomarkers among patients with COPD inform the interpretation of the current findings. Fibrinogen, an acute phase protein elevated in systemic inflammation, has demonstrated an association with all-cause mortality in COPD [7]. A strong association between fibrinogen levels and platelet count has been reported [6]. Interleukin-6, a pro-inflammatory cytokine which has also been associated with increased mortality in COPD [27, 28], induces fibrinogen gene expression [29] and increases thrombopoetin production, which increases circulating platelets [30]. Several inflammatory mediators are known to activate platelets, which can then bind to vascular endothelial cells or adherent leukocytes, perpetuating or destabilizing a plaque [31]. The receptor for one inflammatory mediator, platelet activating factor, which perpetuates platelet activation and is associated with CV morbidity, is upregulated within airway epithelial cells of individuals with COPD suggesting a potential role in COPD with comorbid CV disease [32, 33]. Furthermore, in a cohort of individuals with COPD and stable coronary artery disease undergoing percutaneous coronary intervention, dual antiplatelet therapy with Ticagrelor improved markers of endothelial dysfunction and platelet activation suggesting that these mechanisms may be involved in the morbidity of coincident COPD and CV disease that was observed in this study [34].

A high prevalence of cancer related mortality was present in the lowest platelet count quintile, despite exclusion of patients with known malignancy from SUMMIT, which suggests presence of an underlying comorbidity with high cancer risk, subclinical malignancy or bone marrow suppression that may be driving increased mortality risk in this group. An association between thrombocytopenia and increased cancer mortality has been consistently reported in prior studies [9, 11–13]. Thrombocytopenia is also associated with increased mortality risk when measured at the time of hospital admission for community acquired pneumonia, independent of infection severity [35]. In this population of COPD patients at high risk for respiratory infections, thrombocytopenia in the stable state likely places them at higher risk of thrombocytopenia and poor outcomes during future acute illness.

There are limitations to this study. Few patients were represented in the lowest and highest platelet count groups, which limited the power to make inferences about these groups. The infrequency of mortality events precluded statistical comparison of case-specific mortality between platelet count quintiles. The study sample included moderate COPD patients with CV disease history or risk factors, which may limit generalizability to all COPD patients. Finally, inflammatory biomarkers were not measured in this study, which precluded investigation of correlations between platelet count and inflammatory biomarkers and limited inferences regarding mechanism.

## Conclusions

In conclusion, among patients with moderate COPD and CV disease history or risk factors, there is a U-shaped association between platelet count and all-cause mortality. Higher platelet count was also associated with trends towards increased risk of composite CV disease, although effect estimates for platelet count quintiles and categories were imprecise for all contrasts. Platelet count measured at baseline did not predict future COPD exacerbations.

## Abbreviations

AECOPD: Acute exacerbation of chronic obstructive pulmonary disease
COPD: Chronic obstructive pulmonary disease CV Cardiovascular
FEV_1_: Forced expiratory volume in 1 s
ICS: Inhaled corticosteroid
LABA: Long-acting beta-agonist
mMRC: modified Medical Research Council
Q1: First/lowest quintile
Q3: Third/middle quintile
Q5: Fifth/highest quintile
SUMMIT: Study to Understand Mortality and Morbidity in COPD
